# Autopsy learning module: a tool for assessing self-reflection and practice improvement competency

**DOI:** 10.5116/ijme.5a2b.a8ea

**Published:** 2017-12-11

**Authors:** Mohammad Saud Khan, William Barnett, Amira Gohara, Jacob Torrison, Christian Coletta, Ragheb Assaly

**Affiliations:** 1 Department of Internal Medicine, University of Toledo, OH, USA; 2 Department of Pathology, University of Toledo, OH, USA

**Keywords:** Autopsy, learning module, assessing self-reflection, practice improvement competency

## Introduction

Practice-based learning and improvement is one of the six core competencies listed by the Accreditation Council for Graduate Medical Education (ACGME) for residency programs (post-graduate medical training). ACGME defines practice-based learning and improvement as "an ability to investigate and evaluate the care of patients, appraise and assimilate scientific evidence, and continuously improve patient care based on constant self-evaluation and life-long learning".^1^ Assessing resident (also known as housestaff or a house officer) competency in practice-based learning and improvement is the most challenging of the six competencies as there are very few published tools to assess a resident's performance in this competency. We aim to develop a standardized resource, which could be utilized by residents for learning and self-reflection, and would also provide evaluators with an objective tool for assessing practice-based learning and improvement competency of residents through an approach known as the "Autopsy Learning Module".

Autopsy has long been recognized as an important tool for learning and quality improvement in clinical practice. Physicians often utilize autopsy findings for evaluating the accuracy of their clinical diagnosis, identifying any unsuspected conditions, and establishing a cause of death. To reiterate the importance of autopsy in medical education, we performed a study through a retrospective chart review of 141 autopsies conducted at our institution between January 2011 and May 2015. We measured the level of agreement between the clinical cause of death established by the treating clinician and the cause of death listed by the pathologist after autopsy using Kappa coefficients. We found there was poor agreement between the clinical and autopsy diagnosis, especially among cases of pulmonary embolism and liver cirrhosis, while there was moderate agreement among cases of myocardial infarction. Our findings were consistent with the available literature.^2^ The rate of disagreement between the clinical diagnosis and autopsy findings highlights an opportunity to use an autopsy in a meaningful way as both a learning experience and as a tool for self-reflection.

### The diminishing role of autopsy in medical education

Despite the well-established role of autopsy in learning, institutions have failed to establish it appropriately into the medical curriculum. Hence, the autopsy rates have declined significantly in the past few decades. According to Hull and colleagues, the current autopsy rate is approximately 5% at community hospitals and 10% at academic hospitals, which is far less than approximately the 70% rate at teaching hospitals in the 1960s.^3^ Likewise, at our institution, the yearly autopsy rate is approximately 9.9%. The reason for decline in the autopsy rate is multifactorial. The most frequently cited reasons are physician perception that medical autopsies are unnecessary because they already know the cause of death due to advanced diagnostic tools. Additionally, physician/healthcare provider disinterest and lack of enthusiasm or financial incentives can discourage regularly completed autopsies. Likewise, physician perception that the family is distraught or agitated and would not be willing to consent for autopsy can prevent clinicians from seeking permission to conduct an autopsy.

In academic institutions, residents are closely involved in patient care and have an opportunity to secure consent from a patient's family for an autopsy. Based on our experience and studies in the literature, it is evident that residents lack skills and knowledge for requesting an autopsy. For example, studies on resident perception of autopsy importance and procurement showed that both internal medicine and pathology residents agree that autopsies are an important learning tool, but feel they do not have adequate knowledge and skills about the requisition process and technical aspects of an autopsy.^3^^,^^4^ In addition, Welsh & Kaplan stressed a need for systematic methods of communication between clinicians, pathologists, and families that could improve autopsy rates, support the grieving family, and enhance medical education.^4^

### Autopsy learning module

We realize there is an unmet need for training and educating residents about the autopsy process and implementing measures to improve autopsy rates. In order to assess feasibility of introducing a program, we analyzed the number of monthly autopsies conducted at our hospital for five consecutive years. We found there is a high likelihood that each resident would be a part of this learning experience at least once during their 3-year residency period. Additionally, since each resident is required to complete an intensive care unit (ICU) rotation every year, there is ample opportunity to complete this exercise.

Since building teams for practice-based learning are part of our residency curriculum, the critical care fellow, senior resident, and interns (first-year resident) rotating in the ICU would be responsible to complete an online training before starting the rotation. The training would address the difference between a usual case and coroner case along with how to communicate with the coroner's office. Furthermore, each resident will learn how to introduce the autopsy consent form, discuss the goals of life and end-of-life decisions with the patient's family, break the bad news at the time of demise, obtain consent for autopsy from family members, and educate the families about the process and technical details of an autopsy. After completion of the autopsy, residents would be required to fill out an Autopsy Self-Reflection Forms, in which the resident would document his or her learning experience from participating in the autopsy process. This would include comments from the resident explaining specific points learned from the autopsy that added to the resident's medical knowledge and clinical experience. In addition, the resident can suggest what could have been done differently in the future to improve outcomes. These forms would be included in the portfolio of the resident and evaluated by the program director at the end of the year to assess resident performance. We believe that implementing these measures in the residency curriculum will be a substantial resource for learning and self-reflection for residents, will provide evaluators with a tool for assessing practice-based learning and improvement competency, and will also increase autopsy rates at our institution.

### Lessons learned

Through the development of the Autopsy Learning Module, we learned that in addition to clinical experience, house staff should be given an opportunity to self-reflect on the patients they care for during their training. Furthermore, we have understood that self-reflection resonates within the resident and has a positive effect on their life-long learning practices.

## Conclusions

While resident physicians are in strategic positions to interact with patient families, they often lack the prerequisite skills and experience in the autopsy consent process, which may lead to poor self-assurance. The Autopsy Learning Module allows residents to actively participate in the autopsy process. Through this experience, residents gain confidence in both securing consent from families and communicating with the coroners and pathologists. In addition, a completed module allows program residency directors the tangible means to assess a resident's competency in practice-based learning and improvement.

### Conflict of Interest

The authors declare that they have no conflict of interest.
